# Successful pregnancy using immature oocytes retrieved from resected borderline ovarian tumor: a case report and literature review

**DOI:** 10.1186/s40834-024-00285-9

**Published:** 2024-05-16

**Authors:** Shotaro Higuchi, Tsutomu Miyamoto, Kenji Oka, Hisanori Kobara, Tanri Shiozawa

**Affiliations:** 1 Department of Obstetrics and Gynecology, Shinshu University School of Medicine, 3-1-1 Asahi, Matsumoto, Nagano, 390-8621 Japan; 2 OKA Ladies Clinic, 1-14-1 Shimohigano, Nagano, Nagano, 381-2216 Japan

**Keywords:** Ovarian tumor, Fertility preservation, Immature oocyte, In vitro maturation (IVM), Borderline ovarian tumor

## Abstract

**Background:**

Despite the recent progress of fertility preservation technique, achievement of pregnancy in women with ovarian tumor is still challenging. Here, we report a case of OTO-IVM (ovarian tissue oocyte in-vitro maturation) resulting in a successful delivery.

**Case presentation:**

The patient, a 33-year-old woman with a history of left borderline ovarian tumor (BOT) who underwent left salpingo-oophorectomy three years ago, presented with an enlarged right ovary during infertility treatment, indicating the recurrence of BOT. Because the patient disagreed with curative surgery and normal part-preservation surgery, we eventually performed OTO-IVM. A right salpingo-oophorectomy was first performed. Eight immature oocytes were immediately aspirated not only from visible follicles, but also from entire cortex for invisible follicles, of the removed ovary. In addition, IVM procedure generated six mature oocytes, and were subjected to intracytoplasmic sperm injection (ICSI). Accordingly, three embryos were obtained and cryopreserved. Three months after surgery, hormone replacement therapy was initiated, and a frozen-thawed embryo was transferred, resulting in a successful pregnancy. Although a cesarean section was performed at 36 weeks due to maternal ileus, the baby was delivered without complications.

**Conclusions:**

This report indicates this treatment to be an effective approach for fertility preservation in BOT patients, especially, the importance of collecting oocytes from the entire ovarian cortex was suggested.

## Background

In recent years, the practice of cryopreserving embryos, oocytes, and ovarian tissues has attracted attention as effective ways to preserve fertility for female cancer patients, including those with breast cancer, hematologic malignancies, and other forms of cancer [1]. However, patients with ovarian tumors present specific difficulties because of the anatomic characteristics of these tumors, i.e., the process of ovarian puncture for oocyte retrieval carries the risk of tumor cell dissemination within the abdominal cavity [2]. Additionally, cryopreservation of ovarian tissue raises concerns about potential neoplastic cell contamination, leading to the possibility of tumor re-implantation in the future [3, 4]. Therefore, the development of safe and effective fertility preservation strategies for patients with ovarian tumors is mandatory. One promising approach to address this issue is in vitro maturation of oocytes retrieved ex vivo from ovarian tissue (OTO-IVM) [5]. In OTO-IVM procedures, the surgical resection of the affected ovary is first performed, followed by the retrieval of immature oocytes from the excised ovarian tissue. These immature oocytes are subsequently subjected to in vitro maturation (IVM) to develop into mature oocytes [6], which can be either cryopreserved or used for intracytoplasmic sperm injection (ICSI). Importantly, since oocyte retrieval occurs outside the patient’s body, the risk of tumor cell spillage into the abdominal cavity is eliminated. In this report, we present a unique case of OTO-IVM. The patient first underwent surgical resection of the right ovary which contained both a normal ovarian part and recurrent borderline tumor (BOT), and then immature oocytes were retrieved from the normal part of the resected ovary, especially from its entire ovarian cortex. The immature oocytes were matured by IVM, and used for IVF, resulting in a successful birth. This represents the sixth case reported in the English literature [7–9], and the third case with recurrent BOT. We also performed a literature review to clarify the specific points of the present case.

## Case presentation

**Fig. 1:**
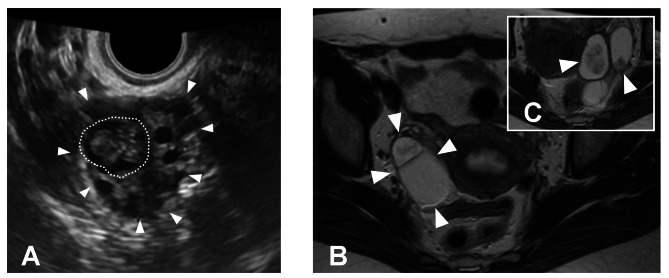
Image findings of the present case. **A**: Transvaginal ultrasonography. Right ovary (arrowhead) including solid part (dotted line). **B, C**: MRI (T2-WI). A multifocal mass with a solid area (arrowhead) was observed in the right ovary (**B**), closely resembling that on preoperative MRI for a left ovarian tumor (**C**, arrowhead)

The patient was a 33-year-old parous woman. When the patient was 30 y.o., she underwent left salpingo-oophorectomy due to a serous borderline tumor, FIGO stage Ia. Three years after the surgery, she started infertility screening and intercourse timing therapy. During the screening and treatment, a 35-mm cystic mass with a solid part in the remaining right ovary was identified, and she was then referred to our hospital. Transvaginal ultrasonography and MRI revealed a cystic mass with solid part in the right ovary (Fig. 1A and B) similar to the previous left ovarian tumor (Fig. 1C). The preoperative diagnosis was a recurrent right ovarian serous borderline tumor after left salpingo-oophorectomy. We first recommended right salpingo-oophorectomy, total hysterectomy, and omentectomy to the patient; however, she and her husband strongly desired to preserve fertility. We then proposed fertility-preserving surgery (FPS) which preserves the normal part of the ovary, since numerous successful cases have already been reported by this procedure [10]. However, they refused FPS due to the risk of recurrence. In addition, they refused ordinary transvaginal oocyte retrieval because of possible tumor dissemination caused by ovarian puncture. Ovarian tissue cryopreservation was also refused due to the risk of tumor reimplantation. Eventually, we devised a method of retrieving oocytes *ex-vivo* from the surgically resected ovary allowing us to cure the tumor and preserve fertility simultaneously. This strategy was approved by the ethics committee of our institute, and the following procedures were performed after obtaining a written consent.

**Fig. 2:**
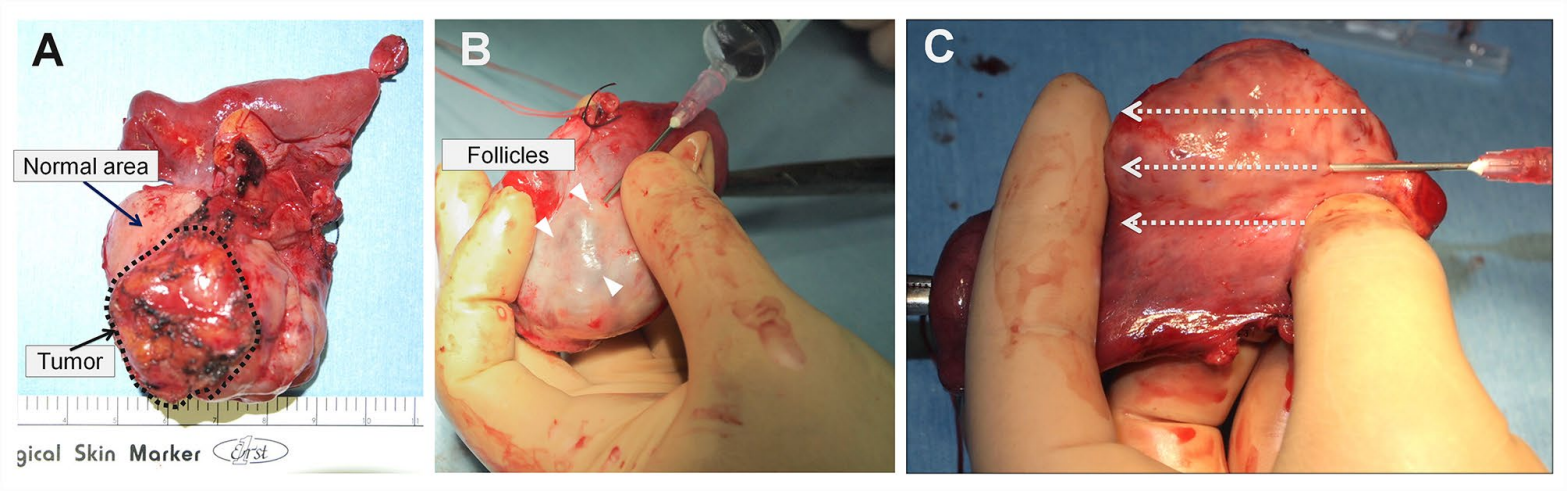
Oocytes retrieval from resected ovary. **A**: The tumor area was identifiable by palpation and ultrasound. **B**: Multiple follicles were observed on the opposite side of the tumor, and visible follicles were first punctured. **C**: The entire ovarian cortex was punctured, even in areas where follicles were not visible

Preoperative blood sampling showed AMH of 4.78 ng/mL, indicating that her ovarian function was preserved. We set the operation day between the 7th to 9th day of menstruation before the predominant follicle appears. However, because the menstruation was delayed, right salpingo-oophorectomy and partial omentectomy were performed on the third day of menstruation. The serum level of progesterone on the operation day was still high, at 3.6 ng/mL. Five thousand units of hCG (Mochida, Japan) were intramuscularly administered 35 h before the expected time of removal of the ovary, according to a previous report describing that the maturation rate of IVM increases when hCG is administered before oocyte retrieval [11]. At that time, six follicular follicles smaller than 10 mm were observed in the right ovary by transvaginal ultrasound. During laparotomy, there was no peritoneal dissemination, and cytology of ascites was negative. In order to minimize the ischemic time, ovarian vessels were amputated as the last step of surgery. We immediately retrieved oocytes from the resected ovary on a workbench in the operating room. The procedure of oocyte retrieval from the resected ovary is shown in Fig. 2. The location of the tumor was carefully determined by palpation and ultrasound (Fig. 2A). In the normal ovarian region, five grossly visible follicles were identified. They were subsequently punctured, and the follicular fluid was aspirated using an 18-gauge needle (Fig. 2B). Then, invisible follicles were retrieved by aspiration of the entire ovarian cortex (Fig. 2C). Accordingly, eight immature oocytes were obtained, and subjected to IVM. We used SAGE In Vitro Maturation Media Kit (CooperSurgical Inc., USA) based on the manufacturer’s instructions. Follicle-stimulating hormone (FSH) 75 IU and human chorionic gonadotropin (hCG) 5000 IU were added to the oocyte maturation medium. IVM resulted in six mature oocytes: two at 24 h, three at 28 h, and one at 52 h. ICSI was then applied for fertilization, and three embryos were obtained. They were cryopreserved by the vitrification method on Day 3 (8-cell Veeck2, 4-cell Veeck2, 5-cell Veeck2).

The postoperative course was uneventful. The pathological diagnosis was a serous borderline tumor of the right ovary, and no additional treatment was given. Three months after the surgery, we planned a frozen-thawed embryo transfer using the same procedure as that of usual IVF-ET. Since the bilateral ovaries had been removed, a hormone replacement cycle was needed. From the third day of menstruation (Day 3), conjugated estrogens (1.25 mg/day, daily) were administered orally and increased to 1.875 mg/day from Day 9. Oral administration of chlormadinone acetate (6 mg/day, daily) was initiated on Day 21. Approximately 58 h after the start of chlormadinone acetate, we conducted a single embryo transfer, 8-cell Veeck2, and confirmed a positive urine hCG two weeks after embryo transfer. Then, conjugated estrogens were switched to transdermal estradiol absorption formulation (1.44 mg, every other day), and chlormadinone acetate was replaced with transvaginal progesterone (600 mg/day, daily). Hormone replacement was discontinued at ten weeks of pregnancy. Although the subsequent pregnancy course was uneventful, the patient was admitted to the hospital due to nausea and vomiting at 36 weeks of gestation and diagnosed with intestinal obstruction. Emergency cesarean section and intestinal obstruction repair were performed. The cause of the bowel obstruction was adhesion of the ileal terminal and sigmoid colon. The postoperative course was uneventful, the newborn’s condition was good (2906-g boy), and no anomaly was observed.

## Discussion and conclusions

Fertility preservation poses unique challenges for patients with BOT. In cases where BOT recurs after unilateral salpingo-oophorectomy, we face a complex decision-making process. In this report, we successfully achieved a live birth using OTO-IVM in a patient with recurrent BOT, indicating that OTO-IVM is a promising technique for patients with various malignancies [1, 5]. While there have been several reports of its successful application since the first reported live birth in 2014 [7], 5 live births have been reported [7–9] (Table 1). Among these reports, this is the third report of BOT.

**Table 1:** List of reported cases of live birth from OTO-IVM

	Maternal age		History	Immature oocytes	MII oocytes after IVM	Maturation rate (%)	Cryopreservation	2PN oocytes after ICSI	Embryo stage	Gestational age	Delivery mode	Weight of baby(g)	Sex of baby
	at OTO-IVM	at delivery											
Prasath [7] (2014)	21	NA	Lt ovary recurrent serous borderline tumor	4	4	100%	embryo	3	cleavage stage	NA	NA	2580	M
Uzelac [8] (2015)	23	26	Lt ovary recurrent mucinous borderline tumor	10	4	40%	embryo	3	cleavage stage	term	NA	3883	M
Segers [9] (2020)	23	26	Hodgkin lymphoma	22	7	32%	oocyte	4	cleavage stage	39w4d	VD	3150	M
	26	27	Uterine Arteriovenous Malformation	13	6	46%	embryo	3	cleavage stage	38w2d	CS	2660	F
	36	42	Breast cancer	8	3	38%	embryo	3	cleavage stage	40w6d	CS	3860	F
present case	34	36	Rt ovary recurrent serous borderline tumor	8	6	75%	embryo	3	cleavage stage	36w6d	CS	2906	M

COC: cumulus-oocyte, VD: vaginal delivery, CS: Cesarean section

Regarding the present OTO-IVM process, we consider that the number of oocytes retrieved is an essential factor for a favorable outcome. In this case, we successfully obtained eight immature oocytes, being more than in a previous report of a live birth [7], and the same as another [8]. In general, it is difficult to retrieve enough oocytes from the resected ovary in the presence of recurrent BOT, since the average number of retrieved oocytes in OTO-IVM without ovarian tumors was reported 14.3 [9]. This observation was also supported in other reports. For example, Segers et al. reported that immature oocytes could not be collected in one patient with BOT among 34 patients who underwent OTO-IVM, including 32 cases with extra-ovarian diseases [12]. Boung et al. also conducted OTO-IVM in 10 cases with or without ovarian tumors, and reported that only 1.5 (mean) immature oocytes were retrieved when ovarian tumors were present, but 14 immature oocytes when tumors were absent [13]. Therefore, we consider that we were able to obtain a large number of oocytes. In order to obtain a greater number of oocytes from the resected ovary, we devised the retrieval approach: we aspirated oocytes not only from visible follicles but also punctured the entire ovarian cortex to aspirate smaller, invisible follicles. The higher yield of oocytes obtained by this approach than the number of follicles observed preoperatively suggests the effectiveness of puncturing the entire ovary. Although there are no reports of OTO-IVM procedures involving puncturing the entire ovarian cortex, we believe that maneuver is effective to retrieve more oocytes. We should take care to avoid puncturing the ovarian tumor when we puncture the ovarian cortex. However, since oocytes were collected from the removed ovary ex vivo, the present method has the advantage that the risk of tumor cells scattering or disseminating into the peritoneal cavity is rare.

We consider that the next important issue is the maturation rate. In our study, we collected eight immature oocytes and six (75%) matured, which were used for ICSI. OTO-IVM typically demonstrated a low maturation rate ranging from 23 to 62% [9, 14–18], whereas maturation rates of IVM for oocytes obtained by transvaginal puncture range from 48 to 67% [19–25]. The maturation rate in the present case was 75%, high enough to be comparable to transvaginal oocyte retrieval. To achieve a high maturation rate, we first minimized the time from resection to oocyte retrieval to avoid ovarian ischemia damage. Thus, the ovarian vessels were amputated at the end of surgery. We immediately retrieved oocytes from the resected ovary with a syringe containing HEPES-buffered human tubal fluid medium on a workbench in the operating room. The follicular fluid containing oocytes was promptly transported from the operating room to embryology laboratory in the same building. Subsequently, embryologists carefully observed the follicular fluid to determine the presence of oocytes. However, previous reports described different methods for managing resected ovaries before oocyte retrieval, with varying impacts on maturation rates [26, 27]. Prasath and Uzelac immersed the resected ovaries in HEPES-buffered human tubal fluid medium and started oocyte retrieval within 20 and 15 min, respectively, after transport from the operating room to embryology laboratory [7, 8]. In contrast, Segers et al. reported that ovaries were transported in a sterile 0.9% saline solution on ice, and transport took up to 3 h before the procedure [9]. However, in Segers’ case, the maturation rate was lower than in other reports. Another study revealed that transporting ovarian tissue on ice had a detrimental effect on maturation when compared with samples that were not transported on ice [26]. We consider that our ability to achieve a maturation rate comparable with that of regular OPU (oocyte pick up)-IVM was due to puncturing them shortly after removal without excessive cooling.

To increase the number of mature oocytes obtained from resected ovaries, some experts suggest performing controlled ovarian stimulation (COS) before surgery and adopting the OTO-COS approach [27–31]. Previous reports stated that the average number of mature oocytes retrieved following oophorectomy in the presence of ovarian tumors with OTO-COS was 11.6 (ranging from 8 to 15 per case) [27–31], whereas for OTO-IVM, it averaged 3.6 (with a range of 3 to 4 per case) [7, 8, 32]. One reason for the higher oocyte retrieval numbers with OTO-COS is that the follicles at the time of retrieval are larger, making it easier to identify the puncture site both visually and with ultrasound guidance [28]. However, ovarian stimulation could potentially lead to ovarian enlargement, increasing the risk of torsion or rupture pre-surgery and complicating the operative procedure [33, 34]. Moreover, the surgery date must be scheduled according to follicular development, making it challenging to secure staff and facilities for the surgery. Further study is needed to perform OTO-COS more safely and effectively. The administration of hCG before oocyte retrieval for IVM remains controversial. Chian et al. reported its potential benefits, including increased rates of maturation, fertilization, and cleavage in IVM [11]. However, now, negative opinions regarding the effectiveness of hCG administration for the success of IVM are predominant [35]. Despite the lack of clear evidence regarding the efficacy of hCG, we decided to administer hCG, expecting its potential contribution to oocyte maturation in the present case.

In this particular case, oocyte retrieval was performed on the third day of the menstrual cycle, with a high progesterone (P4) level of 3.61 ng/mL. Studies focusing on immature oocyte retrieval for fertility preservation in cancer patients have shown no significant differences in the number of retrieved oocytes, maturation rates, or fertilization rates between follicular and luteal phases of the menstrual cycle [36]. Furthermore, the retrieval of immature oocytes has been demonstrated to be feasible even when P4 levels are significantly elevated, as observed in cases of cesarean Sect. [37]. These findings suggest that OTO-IVM may allow for the retrieval of immature oocytes regardless of the menstrual cycle phase, offering the possibility of immediate surgery without the need for waiting periods, thus aiding in fertility preservation.

We reported the sixth case of a live birth resulting from in vitro fertilization using oocytes retrieved from ovarian tissue in an ovarian tumor patient. This approach allows for the preservation of fertility in BOT patients with minimal risk. OTO-IVM provides the potential to obtain multiple oocytes and offers flexibility in the timing of surgery. To maximize the successful utilization of OTO-IVM for ovarian tumors, it is imperative to initiate oocyte retrieval immediately following ovary resection, puncture the entire ovarian cortex, and refrain from excessive ovarian cooling. This approach significantly augments the likelihood of success and is pivotal for ensuring the procedure’s efficacy.

## Data Availability

No datasets were generated or analysed during the current study.

## Abbreviations

OTO-IVM: Ovarian tissue oocyte in-vitro maturation
BOT: Borderline ovarian tumor
ICSI: Intracytoplasmic sperm injection
IVM: In vitro maturation
FPS: Fertility-preserving surgery
OPU: Oocyte pick up
COS: Controlled ovarian stimulation
P4: Progesterone
